# HSP70-mediated regulation of P-glycoprotein SUMOylation suppresses ferroptosis to drive thermoresistance in non-small cell lung cancer

**DOI:** 10.3389/fimmu.2026.1844793

**Published:** 2026-07-21

**Authors:** Jun Wan, Bin Peng, Xiean Ling, Xi Wang

**Affiliations:** 1 Department of Thoracic Surgery, Shenzhen People’s Hospital, The Second Clinical Medical College of Jinan University, The First Affiliated Hospital of South University of Science and Technology, Shenzhen, Guangdong, China; 2 Department of Critical Care Medicine, Shenzhen People’s Hospital, The Second Clinical Medical College of Jinan University,The First Affiliated Hospital of South University of Science and Technology, Shenzhen, Guangdong, China

**Keywords:** ferroptosis, Hsp70, mitochondrial dysfunction, non-small cell lung cancer, oxidative stress, P-gp, sumoylation, thermoresistance

## Abstract

**Objective:**

Hyperthermia is a potential therapy for non-small cell lung cancer (NSCLC); however, cell resistance to hyperthermia remains challenging. This study investigated the role of heat shock protein 70 (HSP70) in regulating small ubiquitin-like modifier modification (SUMOylation) of P-glycoprotein (P-gp) in the resistance of NSCLC cells to hyperthermia.

**Methods:**

Human NSCLC cells (A549) were exposed to hyperthermia and subjected to HSP70 overexpression/knockdown, P-gp silencing, or Erastin/Ferrostatin-1 treatment. Nude mice received inoculation with A549 cells infected with adenoviral vectors overexpressing HSP70 (Ad-HSP70) before hyperthermia. Verapamil was selected as an inhibitor of P-gp *in vivo*. A series of experiments were performed to analyze cellular and molecular changes, including viability, migration, invasion, ferroptosis-related indices, and mitochondrial dysfunction. HSP70-P-gp interaction, SUMOylation, and protein stability were examined using co-immunoprecipitation and cycloheximide assays.

**Results:**

HSP70 overexpression accelerated the malignant properties of A549 cells while restricting ferroptosis, thus driving thermoresistance. Furthermore, activating ferroptosis partially annulled these effects of HSP70 overexpression, whereas inhibiting ferroptosis partially counteracted the suppressive effect of HSP70 knockdown on the thermoresistance of A549 cells. HSP70 interacted with P-gp to modulate its SUMOylation, thereby increasing the stability and levels of P-gp protein. P-gp knockdown partially blocked the effect of HSP70 and restored the sensitivity of A549 cells to hyperthermia. HSP70 suppressed oxidative stress and mitochondrial dysfunction by upregulating P-gp. *In vivo*, HSP70-mediated SUMOylation of P-gp inhibited ferroptosis, promoting tumor growth and thermoresistance.

**Conclusions:**

HSP70 suppresses oxidative stress and mitochondrial dysfunction through SUMOylation of P-gp, thereby inhibiting ferroptosis and promoting thermoresistance in A549 NSCLC cells.

## Introduction

Lung cancer (LC) remains a substantial global health issue, with the highest incidence and mortality rates worldwide (1). According to the statistics available for 2023, around 2 million new LC cases are diagnosed globally each year (2), with poor long-term survival, contributing to its considerable global disease burden (3). As reported, the most common type of LC is non-small cell LC (NSCLC) (4, 5). Recent studies have indicated that hyperthermia is a viable therapeutic strategy for NSCLC (6–8). Hyperthermia leverages the heat sensitivity of cancer cells to enhance the efficacy of other treatments like chemotherapy or radiotherapy, while minimizing damage to surrounding healthy tissues (9). Nonetheless, a significant challenge of hyperthermia is the difficulty in achieving comprehensive tumor eradication, leading to a high recurrence rate as the remaining tumors are prone to adapt to thermal treatment and resume growth (10, 11). Therefore, it is imperative to elucidate the molecular mechanisms underlying thermotolerance in NSCLC to identify potential therapeutic targets and strategies to overcome this resistance.

Ferroptosis, distinct from apoptosis and necrosis, is a programmed cell death pathway featuring iron dependence, lipid peroxidation, ROS accumulation, and mitochondrial dysfunction (12). Targeting ferroptosis represents a novel therapeutic approach for inhibiting tumor progression and overcoming therapeutic resistance in NSCLC (13). For instance, treatment with ferroptosis inducers or manipulation of ferroptosis-regulated genes can suppress tumor cell proliferation and counteract apoptosis-associated drug tolerance in NSCLC (14, 15). Conversely, ferroptosis inhibitors, like chaperonin containing TCP1 subunit 3 and NFS1, have been associated with unfavorable prognosis and enhanced tumor cell proliferation in LUAD (16, 17). However, the regulatory mechanisms of ferroptosis in NSCLC thermotolerance and its potential as a therapeutic target remain to be fully elucidated.

Heat shock proteins (HSPs) are molecular chaperones universally present in cells that maintain protein folding, stability, and cellular homeostasis (18). The HSP70 family represents a highly conserved and inducible subgroup of HSPs (19). HSP70 participates in protein folding and is elevated in reaction to heat stress and toxic insults (20). Notably, in patients with NSCLC, enhanced extracellular HSP70 levels measured preoperatively were associated with early relapse after curative resection (21). Therefore, targeting HSP70 may be a viable strategy for the development of anti-NSCLC treatment.

P-glycoprotein (P-gp) is a 170 kDa glycoprotein and an adenosine triphosphate (ATP)-binding cassette (ABC) transporter, encoded by the ABC subfamily B member 1 gene; it is overexpressed in multidrug-resistant cells and reduces intracellular drug accumulation, thereby conferring resistance to chemotherapeutic drugs, particularly Taxol (22). Recent data have shown that P-gp confers resistance to small-molecule ferroptosis inducers in tumor cells (23). Dysfunction of P-gp triggers mitochondrial stress to sustain ATP production, while prolonged stimulation with P-gp inhibitors leads to the accumulation of ROS and ultimately cell death (24). Notably, HSP70 can suppress ferroptosis and reduce LC recurrence following insufficient radiofrequency ablation by regulating the small ubiquitin-like modifier modification (SUMOylation) of hypoxia-inducible factor-1α (HIF-1α) (25). By contrast, HSP70 depletion induces cancer cell death through endoplasmic reticulum stress and ROS burst (26). In A549 cells, HSP70 re-expression up-regulates P-gp, whereas silencing HSP70 reduces P-gp expression (27). Our GPS-SUMO database analysis predicted multiple SUMOylation modification sites on P-gp. Hence, we hypothesized that HSP70 may stabilize P-gp through SUMOylation, thereby suppressing ferroptosis and ultimately promoting thermotolerance in NSCLC. To date, no studies have reported whether HSP70 mediates thermotolerance in NSCLC through P-gp SUMOylation and the consequent ferroptosis suppression. This study aims to elucidate this mechanism, which may provide novel insights into the molecular basis of thermotolerance and identify potential therapeutic targets to overcome treatment resistance and improve clinical outcomes in NSCLC.

## Materials and methods

### Ethics statement

All animal experiments in this study were reviewed and approved by the Animal Ethics Committee of Shenzhen People’s Hospital (Approval No. AUP-260225-WJ-0094-01). All efforts were made to minimize animal usage and alleviate their suffering during experiments.

### Cell culture

Human NSCLC cell line A549 (CL-0016, Procell, Wuhan, Hubei, China) was cultured in the specific culture medium (CM-0016, Procell) in a 37 °C, 5% CO_2_ incubator.

### Cell grouping and treatment

A549 cells were transfected with overexpression (oe)-HSP70, small interfering (si)-HSP70, si-P-gp, or their respective negative controls (oe-NC, si-NC) utilizing Lipofectamine^®^ 2000 (Invitrogen, Carlsbad, CA, USA) as per the manufacturer’s specifications. All constructs were sourced from GenePharma (Shanghai, China), and the final transfection concentration was 100 ng/μL. Subsequent tests were carried out 24 h following transfection (The results of the cytotoxicity test following plasmid transfection are detailed in **Supplementary Figure S1**; there were no significant differences in cell viability among the groups). A549 cells were subjected to hyperthermia treatment at 40-42 °C for 1 h (28) (Heat group). Untreated cells cultured under normal conditions were regarded as controls (Blank group).

Cells were grouped as follows: (1) Blank group; (2) Heat group; (3) Heat + oe-NC group: cells were transfected with oe-NC and then exposed to hyperthermia; (4) Heat + oe-HSP70 group: cells were transfected with oe-HSP70 and then treated with hyperthermia; (5) Heat + si-NC group: cells were transduced with si-NC and then received hyperthermia treatment; (6) Heat + si-HSP70 group: cells were transduced with si-HSP70 before hyperthermia treatment; (7) Heat + si-HSP70 + Erastin group: cells were transduced with si-HSP70, treated with 5 μM Erastin (a ferroptosis agonist; HY-15763, MCE, Monmouth Junction, NJ, USA) for 24 h, followed by hyperthermia (29); (8) Heat + si-HSP70 + DMSO group: cells were transduced with si-HSP70 and treated with the equivalent volume of dimethyl sulfoxide (DMSO) to Erastin for 24 h, followed by hyperthermia; (9) Heat + oe-HSP70 + si-NC group: cells were co-transfected with oe-HSP70 and si-NC, followed by hyperthermia; (10) Heat + oe-HSP70 + si-P-gp group: cells were co-transfected with oe-HSP70 and si-P-gp, followed by hyperthermia.

### Cell counting kit-8 assay

Cell viability was evaluated using a CCK-8 assay kit (WH1199, Wei’ao Biotech Ltd., Shanghai, China). Briefly, 100 μL of cell suspension (5 × 10^4^ cells/mL) was plated into 96-well plates, and 10 μL of CCK-8 reagent was introduced to each well for a 2-h incubation. Absorbance at 450 nm was determined with a Multiskan FC microplate reader (51119080, Thermo Fisher Scientific, Waltham, MA, USA). Meanwhile, cell viability was computed according to the formula: Cell viability (%) = [(A_experimental group_ - A_blank group_)/(A_control group_ - A_blank group_)] × 100%.

### Transwell assay

Cell invasion was assessed using Transwell chambers precoated with Matrigel matrix (BD Biosciences, San Jose, CA, USA), while cell migration was evaluated using Transwell chambers without Matrigel coating. Prior to the assay, cells were cultivated in serum-free medium for 24 h and subsequently resuspended in medium comprising 1% fetal bovine serum (FBS). Following this, 1 × 10^5^ cells were plated into the upper chamber, and 600 μL of complete medium supplemented with 10% FBS was introduced to the lower chamber. Following 24-h incubation, non-migrated or non-invaded cells in the upper membrane were removed with a cotton swab. Thereafter, cells were immobilized with 4% paraformaldehyde and dyed with hematoxylin (517-28-2, MACKLIN, Shanghai, China). Five random view fields (top, bottom, left, right, and center) were selected for observation, and the number of cells was counted using Image Pro Plus 6.0 (Media Cybernetics, Rockville, MD, USA), with the results averaged.

### Lactate dehydrogenase assay

LDH activity in the culture supernatant was measured utilizing a colorimetric LDH cytotoxicity assay kit (A020-1-2, Nanjing Jiancheng Bioengineering Institute, Nanjing, Jiangsu, China). Absorbance values were documented at 490 nm with a microplate reader to evaluate cell damage.

### Western blot

Total cellular protein was extracted utilizing a protein extraction kit (P0033, Beyotime, Shanghai, China), and protein concentration was ascertained with a bicinchoninic acid (BCA) protein assay kit (P0009, Beyotime). Following electrophoresis, membrane transfer, and blocking, membranes were cultivated overnight at 4 °C with the below primary antibodies: HSP70 (1:1000, ab2787, Abcam, Cambridge, UK), glutathione peroxidase 4 (GPX4) (1:1000, ab125066, Abcam), solute carrier family 7 member 11 (SLC7A11) (1:2000, ab307601, Abcam), and P-gp (1:500, 22336-AP, Proteintech, Wuhan, Hubei, China). After rinsing, membranes were incubated with a secondary antibody immunoglobulin G (IgG) (1:1000, ab205718, Abcam) at 37 °C for 1 h. Protein bands were visualized using a chemiluminescence kit (ECL Plus, Life Technology, Foster City, CA, USA), and grayscale analysis was executed by applying Image-Pro Plus 6.0 (Media Cybernetics, Silver Spring, MD, USA). Glyceraldehyde-3-phosphate dehydrogenase (GAPDH) (1:5000, ab8245/ab181602, Abcam) was used as the internal reference.

### Co-immunoprecipitation

Potential SUMOylation sites of P-gp were predicted using the GPS-SUMO database (https://sumo.biocuckoo.cn/). We constructed the wild-type plasmid P-gp-WT and the corresponding mutant plasmid P-gp-MUT (GenePharma; lysine [K] residues at positions 347, 410, and 413—which had high confidence thresholds—were mutated to arginine [R] residues [the lysine codons AAG or AAA were mutated to the arginine codons AGG or CGG]). Cellular total proteins were extracted, and protein concentrations were examined with a BCA kit (Solarbio, Beijing, China) and adjusted to a consistent level. The supernatant was incubated overnight at 4 °C with anti-P-gp antibody (1:300, 22336-AP, Proteintech) or IgG antibody (1:300, ab172730, Abcam). Afterwards, the protein-antibody complexes were shifted to a centrifuge tube and cultivated with protein A/G agarose under 1-h rotation in a closed container. After centrifugation of elution agarose at 800 × g for 3 min at 4 °C, the immunoprecipitates were boiled, and next subjected to western blot experiments. Protein interactions were analyzed via reaction with antibodies against SUMO1 (1:800, 10329-1-AP, Proteintech), SUMO2/3 (1:600, 11251-1-AP, Proteintech), HSP70 (1:1000, ab2787, Abcam), and P-gp (1:500, 22336-AP, Proteintech).

### Protein stability assay

To assess protein stability, A549 cells were plated into 24-well plates and cultivated for 24 h, prior to transfection with the indicated plasmids. After cells were treated with 20 μg/mL cycloheximide (CHX, HY-12320, MCE) for 3, 6, and 9 h, cell precipitates were collected and tested by western blot.

### JC-1 fluorescence detection

Mitochondrial membrane potential (MMP) was detected using the JC-1 probe (Thermo Fisher Scientific) following the manufacturer’s manuals. Cells were plated at 5 × 10^3^ cells/well in 96-well plates and grown overnight, and then stained with JC-1 solution (10 µM) at 37 °C for 1 h without light. After the culture solution was taken out, the petri dish was rinsed twice with 1 × phosphate-buffered saline (PBS), and fluorescence was examined by means of a fluorescence microscope (Olympus BX53, Tokyo, Japan).

### Assessment of mitochondrial DNA

Total DNA was separated from A549 cells *via* the FlexiGene DNA Kit (Qiagen, Shanghai, China) following the manufacturer’s protocols. An Oligonucleotide primer sequence probe specific for mtDNA were designed as follows: mtDNA F: TGCTAGCCGCAGGCATTAC; R: GGGTGCCCAAAGAATCAGAAC. Real-time fluorescence quantitative polymerase chain reaction (qPCR) was performed to ascertain mtDNA content using Hieff qPCR SYBR Green Master Mix (Yeasen, Shanghai, China), and relative gene expression was calculated using the 2^-ΔΔCt^ technique. Nuclear DNA served as an internal control (F: CTTCCCCACTGGCCTCAAG; R: CCAAAACCCAGTGATCCAGC).

### Determination of ATP

A549 cells were fully lysed and centrifuged at 10000 g for 5 min at 4 °C. The supernatant was harvested for storage on ice. Intracellular ATP levels were tested using an ATP quantitative bioluminescence assay kit (Beyotime) on a multi-functional microplate reader SpectraMax M2e (Molecular Devices, Sunnyvale, CA, USA). All experiments were independently replicated thrice.

### Determination of mitochondrial respiratory oxygen consumption

The Seahorse XF24 extracellular flux analyzer (Seahorse Bioscience, Billerica, MA, USA) was utilized to detect the oxygen consumption rate (OCR). Specifically, A549 cells (4 × 10^4^ cells/well) were introduced in XFe96 cell culture microplates and incubated overnight. The mitochondrial stress test assay included the following compounds: oligomycin (final concentration 1 μM; port A), FCCP (final concentration 0.5 μM; port B), as well as a mixture of rotenone and antimycin A (both at a final concentration of 0.5 μM; port C). Thereafter, the assay was carried out using a Seahorse XFe96 cellular energy metabolism analyzer (Agilent, Santa Clara, CA, USA). The Wave Desktop and Report Generator software (Agilent) was applied to view and analyze the results. The experiment was replicated in triplicate.

### Experimental animal

A total of 30 BALB/c nude mice (4–6 weeks old) (Beijing Vital River Laboratory Animal Technology Co., Ltd., Beijing, China) were reared under a sterile circumstance at 26-28 °C with a 40-60% relative humidity and a 12-h light/dark cycle, with free access to food and water.

### Animal treatment and grouping

A total of 2 × 10^6^ A549 cells were subcutaneously injected into the axillary region of the right forelimb in BALB/c nude mice to trigger *in vivo* tumorigenesis. Tumor volume was examined with a vernier caliper, and once the volume reached 50 mm^3^, mice were randomly assigned to groups for processing. Whole body hyperthermia was conducted in a thermally regulated, well-ventilated chamber heated by infrared rays (Blast Co., Ltd., Tokyo, Japan). The rectal temperature was observed and sustained at 40-42˚C, with heat shock administered for 1 h, once weekly for four consecutive weeks (28). Beginning in the second week after hyperthermia initiation, mice received tail vein injections of the P-gp inhibitor verapamil (25 mg/kg; HY-14275, MCE) thrice per week for three weeks (30), wherein verapamil was dissolved sequentially in 10% DMSO, 40% polyethylene glycol 300, 5% Tween-80, and 45% saline. The mice receiving inoculation of untreated A549 cells to induce tumorigenesis *in vivo* were designated as the control (LC group).

Tumor-bearing nude mice were randomly allocated into groups (n = 6 per group): (1) LC group; (2) Heat group: nude mice received inoculation of untreated A549 cells prior to hyperthermia treatment; (3) Heat + Ad-NC group: nude mice underwent treatment with Ad-NC-transduced A549 cells prior to hyperthermia treatment; (4) Heat + Ad-HSP70 group: nude mice received inoculation of A549 cells transduced with Ad-HSP70 (an adenoviral vector commonly used for gene overexpression *in vivo* and *in vitro*, which achieves transient high expression of HSP70 by infecting target cells or tissues) prior to hyperthermia treatment; (5) Heat + Ad-HSP70 + Verapamil group: mice received inoculation with Ad-HSP70-transduced A549 cells, followed by treatment with verapamil and hyperthermia; (6) Heat + Ad-HSP70 + Vehicle group: mice received inoculation with Ad-HSP70-transduced A549 cells, followed by treatment with vehicle (the same volume as verapamil) and hyperthermia. Tumor tissues were harvested 24 h after the final intervention. Portions of each tumor tissue were immobilized in 4% paraformaldehyde (Solarbio) for 24 h, dehydrated in ethanol, cleared in xylene, embedded in paraffin, sectioned at 5 μm, and deparaffinized to water for immunofluorescence, immunohistochemistry (IHC), and terminal deoxynucleotidyl transferase-mediated dUTP nick end-labeling (TUNEL) staining. The remaining tumor tissues were made into tissue homogenate for reverse transcription-quantitative polymerase chain reaction (RT-qPCR), western blot, and kit detection. Adenoviral vector (Ad-HSP70) and its control (Ad-NC) were acquired from GENECHEM Co., Ltd. (Shanghai, China).

### RT-qPCR

Total RNA was isolated from cells using TRIzol reagent (Invitrogen) guided by the manufacturer’s protocol. Total RNA was reverse transcribed into complementary DNA using the PrimeScript RT reagent kit (Takara, Dalian, China). qPCR was implemented utilizing SYBR Green II (Takara) on an ABI PRISM 7900 Sequence Detection System. PCR procedures were as below: pre-denaturation at 95 °C for 5 min, followed by 40 cycles of denaturation at 95 °C for 15 s, annealing at 60 °C for 20 s, and extension at 72 °C for 35 s. GAPDH was used as the internal reference, and data were analyzed using the 2^-ΔΔCt^ technique. Primer sequences (synthesized by Sangon Biotech, Shanghai, China) are illustrated in **Table 1**.

**Table 1:** Primer sequences.

Gene	Forward (5’-3’)	Reverse (5’-3’)
HSP70	GTTTCGGTTCCTTGTTTCTATACTG	TTTCTACCTCCCAATGTCGTGT
P-gp	TGCTGGAGCGGTTCTACG	ATAGGCAATGTTCTCAGC AATG
GAPDH	CATCATCCCTGCCTCTAC TGG	GTGGGTGTCGCTGTTGA AGTC

### Detection of Fe^2+^, glutathione, malondialdehyde, superoxide dismutase, and ROS levels

Cells or tumor tissue samples were collected, and the concentrations of Fe^2+^, GSH, MDA, SOD, and ROS were examined using the iron assay kit (ab83366, Abcam), GSH assay kit (69-80233, MSK, Shanghai, China), MDA assay kit (ab233471, Abcam), SOD assay kit (S0101M, Beyotime), and ROS assay kit (ab186029, Abcam), in accordance with the manufacturers’ schemes.

### Immunofluorescence

Lipid peroxidation levels (green signal at 484/510 nm and red signal at 581/610 nm) were assessed using the lipid peroxidation sensor BODIPY™581/591 C11 (D3861; Thermo Fisher Scientific) following instructions. Images were collected under a fluorescence microscope (Leica DMI6000B, Weztlar, Germany).

### IHC

Paraffin sections of tumor tissues were rinsed thoroughly with PBS and subsequently subjected to antigen retrieval by boiling in 0.01 M sodium citrate buffer (Sigma-Aldrich, St Louis, MO, USA). Afterwards, sections were treated with 3% H_2_O_2_ at room temperature for 15 min before blocking with 5% goat serum (Solarbio) for 30 min. Sections were cultured in the presence of primary antibodies, including rabbit anti-Ki-67 (0.5 µg/mL, ab15580, Abcam), rabbit anti-Wilms’ tumor 1-associating protein (1:100, ab155655, Abcam), and rabbit anti-GLUT-1 (1:200, ab115730, Abcam) all night at 4 °C. Subsequently, goat anti-rabbit IgG H&L (HRP) (1:1000, ab6721, Abcam) was added to sections for 2-h cultivation at room temperature, with color developed using 3,3′-diaminobenzidine (Solarbio). After counterstaining and mounting, sections were viewed and imaged under a light microscope. The percentage of positive cells was quantified utilizing Image J software (National Institutes of Health, Bethesda, MD, USA).

### Statistical analysis

All data were statistically analyzed and plotted using GraphPad Prism 9.5 (GraphPad Software, Inc., San Diego, CA, USA). Data were expressed as mean ± standard deviation (SD). Data comparisons between two groups were made using *t* tests. Multiple group comparisons were conducted using one-way analysis of variance (ANOVA), followed by Tukey’s multiple comparisons test. A two-tailed *P* value < 0.05 was considered statistically significant.

## Results

### Elevated HSP70 expression inhibits ferroptosis to promote thermotolerance of NSCLC cells

**Figure 1:**
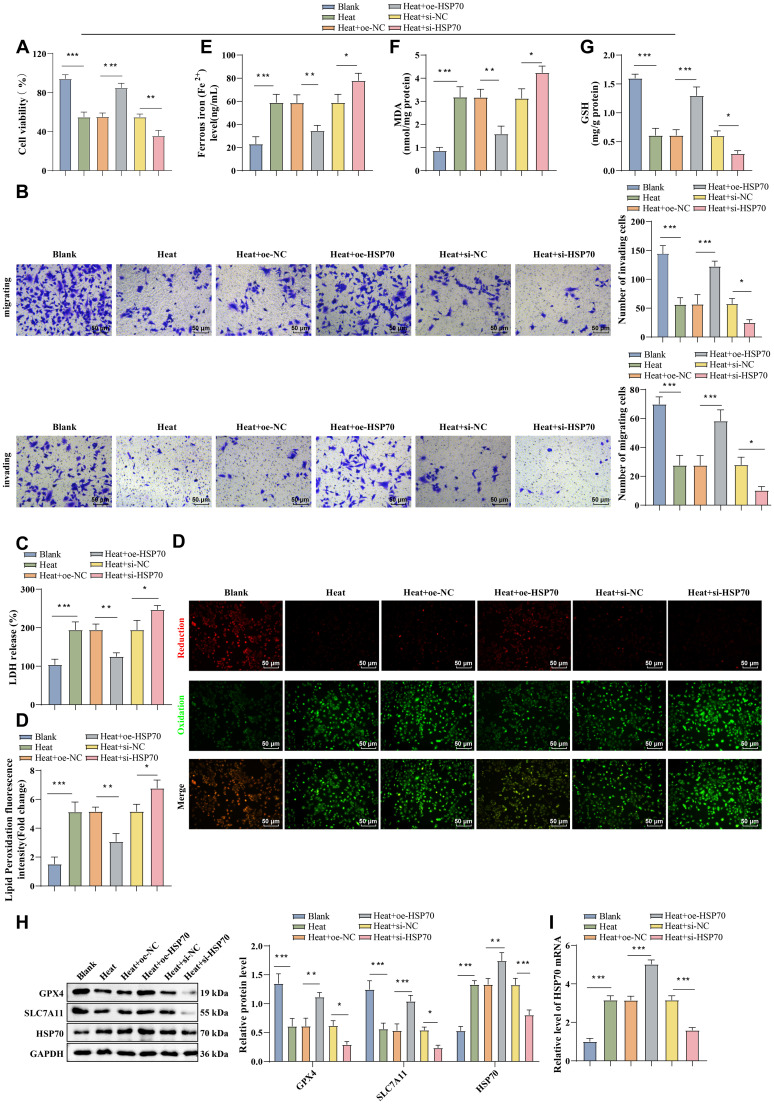
High expression of HSP70 suppresses ferroptosis to promote the resistance of NSCLC cells to hyperthermia. **(A)** Cell viability assessed by CCK-8 assay; **(B)** Cell invasion and migration assessed by Transwell assay; **(C)** Cell death measured by LDH assay; **(D)** Lipid peroxidation levels detected by BODIPY staining; **(E–G)** Intracellular Fe^2+^, MDA, and GSH levels measured using commercial kits; **(H)** Expression of ferroptosis-related proteins (GPX4 and SLC7A11) and HSP70 assessed by western blot; **(I)** HSP70 expression in cells measured by RT-qPCR. All cell experiments were independently repeated three times. Data were presented as mean ± SD. Comparisons among groups were performed using one-way ANOVA, followed by Tukey’s multiple comparisons test. *P < 0.05, **P < 0.01, ***P < 0.001.

To clarify the effects of hyperthermia on NSCLC, A549 cells were initially cultured *in vitro* and subjected to hyperthermia treatment (40-42 °C for 1 h), followed by assessment based on three dimensions: cell viability, invasive and migratory capacity, and ferroptosis characteristics. The results showed that hyperthermia treatment led to significantly suppressed A549 cell viability (*P* < 0.01; **Figure 1A**), reduced migration and invasion (both *P* < 0.01; **Figure 1B**), and markedly increased A549 cell death (*P* < 0.01; **Figure 1C**), elevated levels of lipid peroxidation (the core hallmark of ferroptosis) (*P* < 0.001; **Figure 1D**), raised intracellular Fe^2+^ and MDA levels, and lowered GSH contents (all *P* < 0.001; **Figures 1E–G**). In addition, hyperthermia treatment also diminished the expression of GPX4 and SLC7A11 (ferroptosis-related proteins) (both *P* < 0.01; **Figure 1H**). The results indicate that hyperthermia depresses proliferative activity, migration, and invasion, while driving ferroptosis in A549 cells.

RT-qPCR and western blot further clarified that cellular HSP70 expression was markedly up-regulated following hyperthermia (*P* < 0.001; **Figures 1H, I**), illustrating that HSP70 may act as a stress response molecule for hyperthermia treatment and participate in the regulation of hyperthermia-induced ferroptosis in NSCLC. Next, to ascertain the role of HSP70 in hyperthermia-induced ferroptosis, we engineered oe-HSP70/si-HSP70 to overexpress/knock down HSP70 expression prior to hyperthermia treatment. The results showed that oe-HSP70 transfection in heat-treated cells significantly enhanced HSP70 expression (*P* < 0.01; **Figures 1H, I**), reduced cell death, lipid peroxidation, intracellular Fe^2+^ and MDA levels, augmented GSH, GPX4, and SLC7A11 levels (all *P* < 0.01; **Figures 1C–H**), and boosted cell viability as well as migratory and invasive capacity (all *P* < 0.01; **Figures 1A, B**), highlighting that overexpression of HSP70 impeded ferroptosis and potentiated resistance to hyperthermia in A549 cells. Additionally, compared to the Heat + si-NC group, cells in the Heat+si-HSP70 group showed significantly down-regulated HSP70 expression (*P* < 0.01; **Figures 1H, I**), further increased ferroptosis (all *P* < 0.05; **Figures 1D–H**), along with suppressed cell viability, invasiveness, migration (all *P* < 0.05; **Figures 1A, B**), suggesting that HSP70 knockdown further facilitated hyperthermia-induced ferroptosis in A549 cells and enhanced the antitumor effects of hyperthermia.

### Activation of ferroptosis partially reverses the promotion effect of HSP70 on the thermoresistance of NSCLC cells

**Figure 2:**
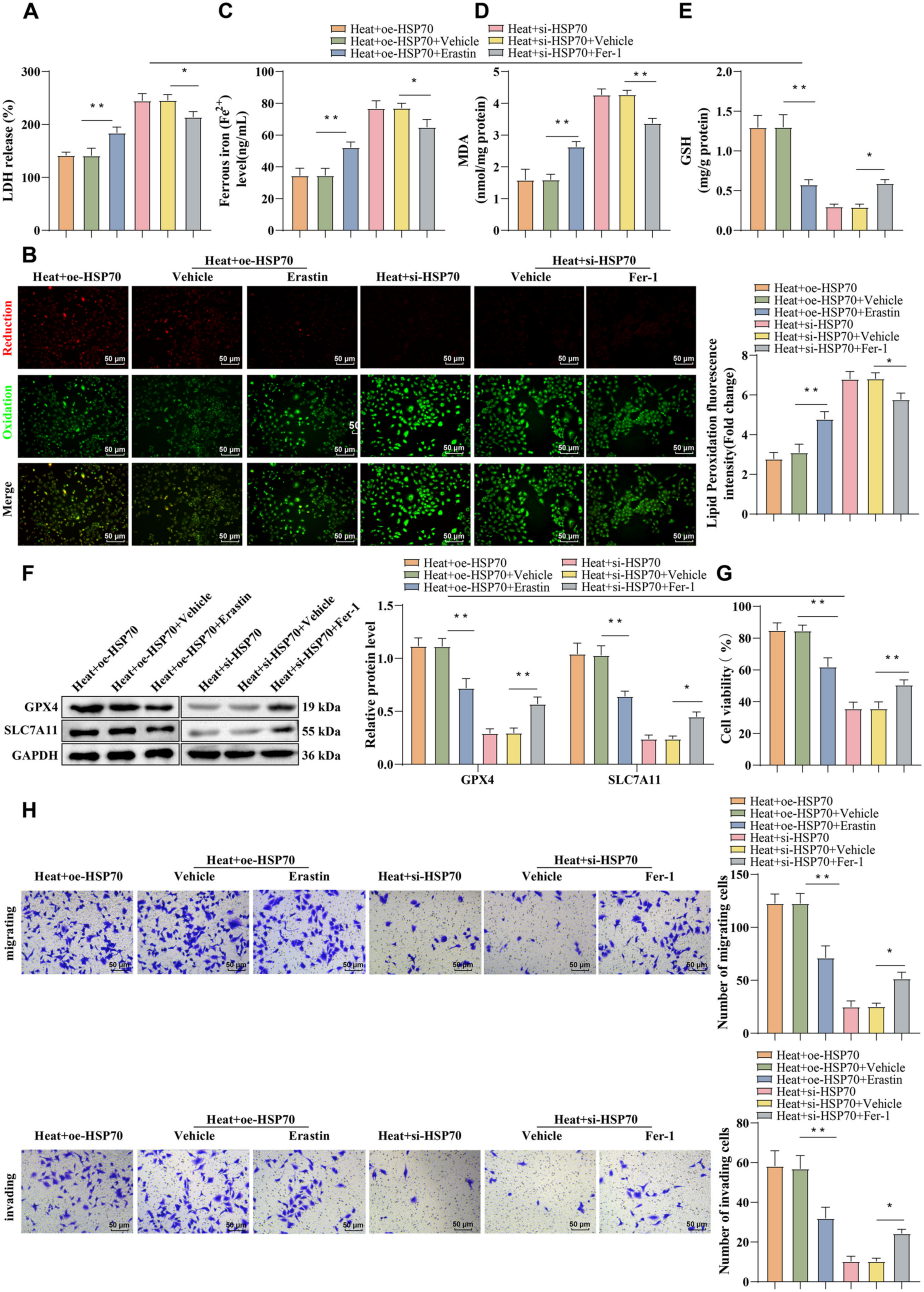
Activation of ferroptosis partially counteracts the promoting effect of HSP70 on the resistance of NSCLC cells to hyperthermia. **(A)** LDH detection of cell death; **(B)** BODIPY staining to detect lipid peroxidation; **(C–E)** Test kits to measure levels of Fe^2+^, MDA, and GSH in cells; **(F)** Western blot to detect the expression of ferroptosis-related proteins (GPX4 and SLC7A11) in cells; **(G)** CCK-8 detection of cell viability; **(H)** Transwell assay of cell invasion and migration. Cell experiments were replicated three times. Data were expressed as mean ± SD. One-way ANOVA was implemented for multi-group data analysis. Tukey’s multiple comparison test was conducted for the *post hoc* test. * *P* < 0.05, ** *P* < 0.01.

To determine whether HSP70 influences A549 cell thermotolerance by regulating ferroptosis, we treated HSP70-overexpressing/knockdown A549 cells with the ferroptosis activator (Erastin) or the ferroptosis inhibitor (Ferrostatin-1) prior to hyperthermia treatment and validated pathway dependence based on three aspects: ferroptosis characteristics, cell viability, and cell phenotypes. The results showed that, relative to the Heat + oe-HSP70 + Vehicle group, the Heat + oe-HSP70 + Erastin group exhibited increased A549 cell death, elevated lipid peroxidation, higher Fe^2+^ and MDA levels, decreased GSH contents, and down-regulated GPX4 and SLC7A11 (all *P* < 0.01) (**Figures 2A–F**), along with markedly curbed cell viability, migration, and invasion (all *P* < 0.01) (**Figures 2G, H**). These results indicated that activating ferroptosis partially abrogated HSP70 overexpression-induced thermotolerance in NSCLC cells, hinting ferroptosis as a key downstream pathway in HSP70-mediated thermotolerance of NSCLC cells (all P < 0.05). Compared with the Heat + si-HSP70 + Vehicle group, the Heat + si-HSP70 + Fer-1 group had reduced ferroptosis in A549 cell (**Figures 2A–F**), significantly decreased cell viability, migration, and invasion (all *P* < 0.05) (**Figures 2G, H**), suggesting that inhibiting ferroptosis partially annulled the enhancing effect of HSP70 knockdown on the sensitivity of A549 cells to hyperthermia. These results indicate that ferroptosis is a core downstream mechanism by which HSP70 regulates the thermotolerance of NSCLC cells, providing direct experimental evidence for targeting the HSP70-ferroptosis pathway to reverse thermoresistance.

### HSP70 enhances P-gp protein stability by regulating SUMOylation of P-gp

**Figure 3:**
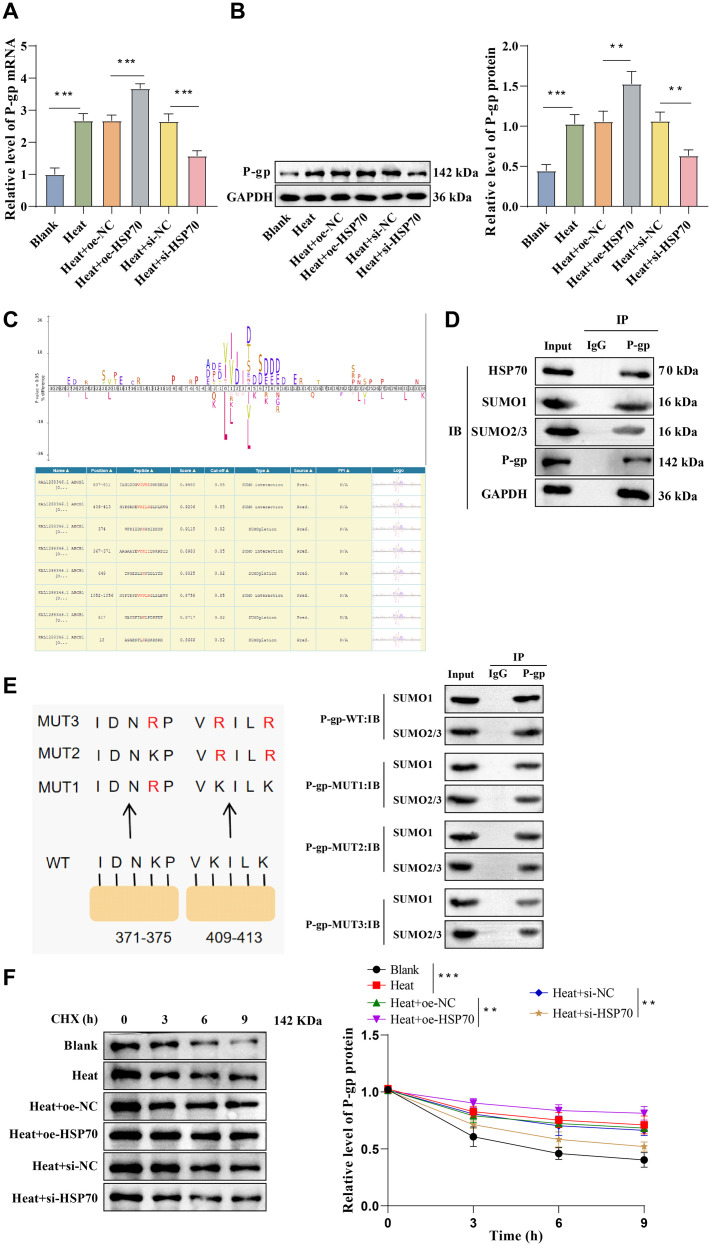
HSP70 enhances P-gp protein stability by regulating SUMOylation of P-gp. **(A, B)** P-gp expression evaluated by RT-qPCR and western blot; **(C)** SUMOylation sites of P-gp predicted using the online GPS-SUMO database; **(D)** Interaction between P-gp and HSP70 and SUMOylation levels of P-gp assessed by Co-IP assay; **(E)** The SUMOylation levels of wild-type and mutant P-gp measured with Co-IP assay; **(F)** P-gp protein stability evaluated by CHX chase assay. All cell experiments were independently repeated three times. Data were presented as mean ± SD. Comparisons among multiple groups were performed using one-way ANOVA, followed by Tukey’s multiple comparisons test. **P < 0.01, ***P < 0.001.

Previous research has reported that P-gp suppresses ferroptosis and drives survival in cancer cells (23). To elucidate whether HSP70 influences ferroptosis by mediating P-gp, we first examined P-gp expression in cells underwent different treatments. Compared with the Blank group, P-gp expression was up-regulated in the Heat group; relative to the Heat + oe-NC group, P-gp expression was further increased in the Heat + oe-HSP70 group, whereas it was diminished in the Heat + si-HSP70 group versus the Heat + si-NC group (all *P* < 0.01; **Figures 3A, B**), revealing that HSP70 could up-regulate P-gp expression under hyperthermia treatment. In addition, a prior study reported that HSP70 regulated the SUMOylation of target proteins to hinder ferroptosis (25). Through prediction using the GPS-SUMO database, we observed that P-gp contained multiple SUMOylation sites (**Figure 3C**), demonstrating that P-gp may be a target of HSP70-mediated SUMOylation. Co-IP results confirmed an interaction between HSP70 and P-gp and demonstrated the SUMOylation of P-gp (*P* < 0.01; **Figure 3D**). Afterward, we constructed P-gp-WT and P-gp-MUT vectors (P-gp-MUT1: K374R; P-gp-MUT2: K410R and K413R; P-gp-MUT3: K374R, K410R, and K413R). The results showed that compared with the P-gp-WT group, SUMOylation level was prominently reduced in the P-gp-MUT1/2/3 groups, with the most significant reduction detected in the P-gp-MUT3 group (**Figure 3E**). Additionally, to verify the effect of SUMOylation on P-gp stability, we performed a CHX assay to detect P-gp degradation. The data revealed that P-gp degradation was reduced and protein stability increased in the Heat group or the Heat + oe-HSP70 group compared with the Blank group or the Heat + oe-NC group, respectively. However, HSP70 knockdown accelerated P-gp degradation and reduced its protein stability (all *P* < 0.01; **Figure 3F**). In summary, these results indicate that HSP70 modulates the SUMOylation of P-gp by interacting with P-gp, thereby enhancing the stability and levels of P-gp protein.

### P-gp knockdown promotes ferroptosis, thus partially reversing HSP70-induced thermoresistance in NSCLC cells

**Figure 4:**
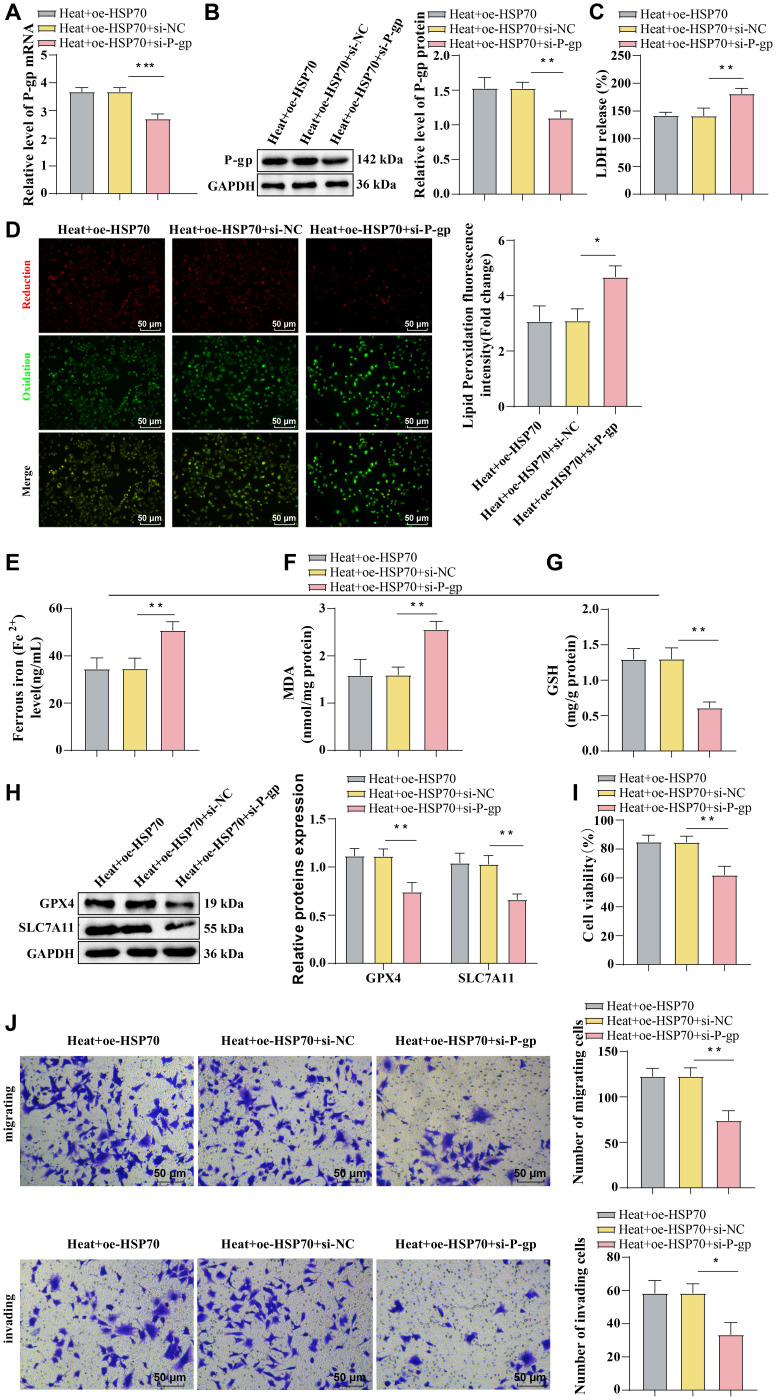
Knockdown of P-gp facilitates ferroptosis, which partially reverses the promoting effect of HSP70 on the resistance of NSCLC cells to hyperthermia. **(A, B)** RT-qPCR and western blot to evaluate P-gp expression in cells; **(C)** LDH to assess cell death; **(D)** BODIPY staining to examine lipid peroxidation; **(E–G)** Test kit to detect Fe^2+^, MDA, and GSH levels in cells; **(H)** Western blot to assess ferroptosis-related proteins (GPX4 and SLC7A11) in cells; **(I)** CCK-8 to examine cell viability; **(J)** Transwell to assess cell invasion and migration. Cell experiments were repeated thrice. Data were exhibited as mean ± SD. One-way ANOVA was utilized for data comparisons among multiple groups, and Tukey’s multiple comparison test was employed for the *post hoc* test. * *P* < 0.05, ** *P* < 0.01, *** *P* < 0.001.

To determine whether P-gp is a key downstream effector molecule regulated by HSP70 in mediating thermotolerance in NSCLC cells, we further transfected A549 cells with oe-HSP70 and si-P-gp prior to hyperthermia treatment. The results demonstrated that in comparison to the Heat + oe-HSP70 + si-NC group, cells in the Heat + oe-HSP70 + si-P-gp group exhibited notably suppressed P-gp expression (both *P* < 0.01) (**Figures 4A, B**), increased cell death, and markedly raised lipid peroxidation, augmented intracellular Fe^2+^ and MDA levels, declined GSH content, down-regulated levels of ferroptosis-associated markers (GPX4 and SLC7A11) (all *P* < 0.05) (**Figures 4C–G**). Meanwhile, as compared to the Heat + oe-HSP70 + si-NC group, the Heat + oe-HSP70 + si-P-gp group presented abated cell viability, migration, and invasion (all *P* < 0.05) (**Figures 4H–J**). These results demonstrate that P-gp knockdown effectively nullifies the repressive effect of HSP70 overexpression on ferroptosis and restores the ferroptotic response induced by hyperthermia. In this regard, P-gp is a key downstream molecule in HSP70-mediated thermoresistance and that P-gp knockdown partially blocks the protective effect of HSP70 and restores the sensitivity of NSCLC cells to hyperthermia.

### HSP70 inhibits oxidative stress and mitochondrial dysfunction by up-regulating P-gp protein levels

**Figure 5:**
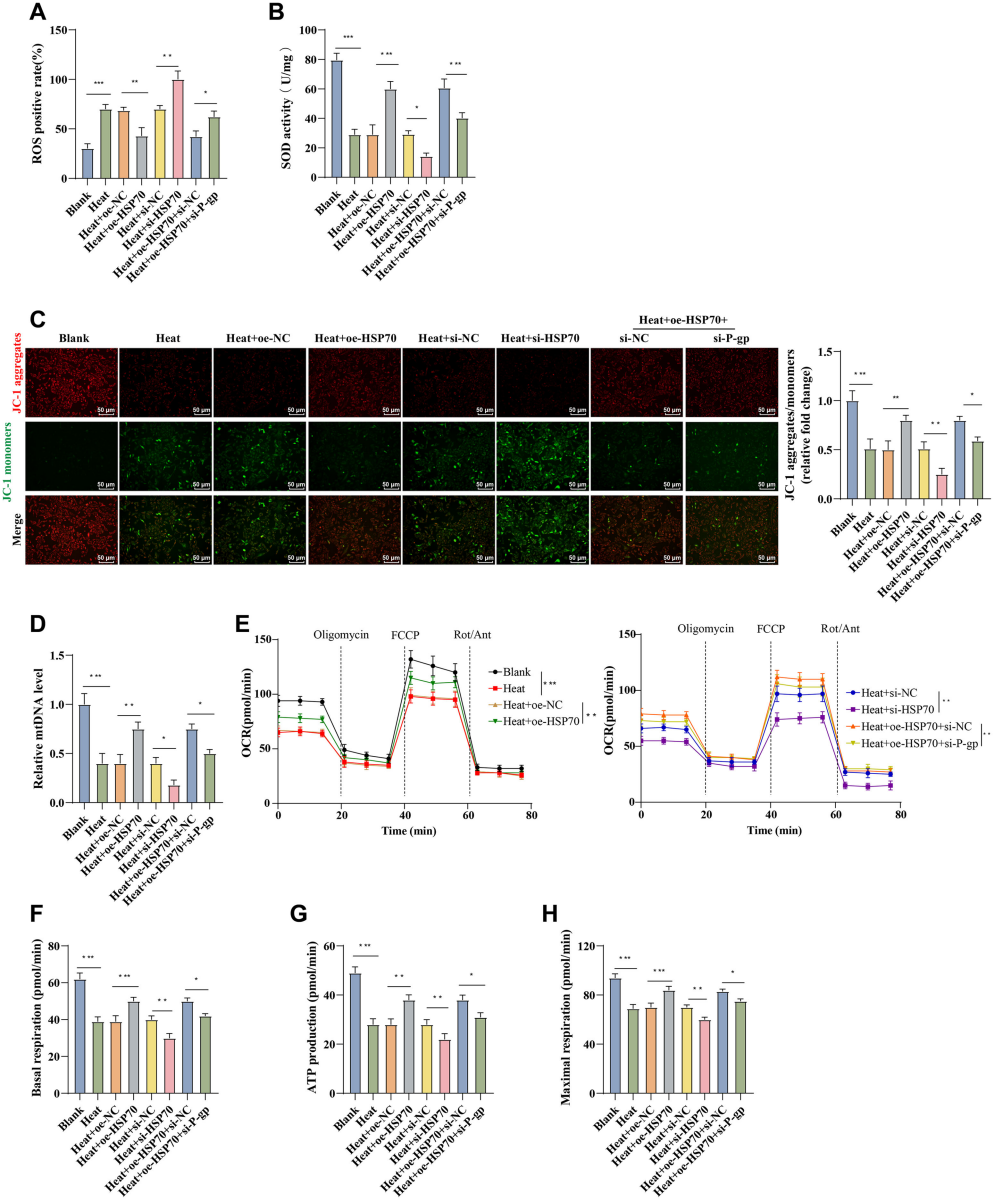
HSP70 suppresses oxidative stress and mitochondrial dysfunction by upregulating P-gp. **(A, B)** Measurement of intracellular ROS and SOD levels using commercial kits; **(C)** Assessment of MMP assessed by JC-1 fluorescent probe; **(D)** Measurement of mtDNA levels; **(E–H)** Real-time monitoring curves of OCR in cells, along with quantitative analyses of basal respiration, ATP synthesis, and maximal respiration levels. All cell experiments were independently repeated three times. Data were presented as mean ± SD. Comparisons among groups were performed using one-way ANOVA, followed by Tukey’s multiple comparisons test. *P < 0.05, **P < 0.01, ***P < 0.001.

Current research has shown that P-gp dysfunction triggers mitochondrial stress to generate ATP, and prolonged stimulation with P-gp inhibitors leads to mitochondrial ROS accumulation and ultimately cell death (24). To further investigate whether HSP70 regulates oxidative stress and mitochondrial function through P-gp, we assessed oxidative and mitochondrial parameters in A549 cells. Compared with the Blank group, the Heat group showed elevated ROS levels and reduced SOD activity (both *P* < 0.01; **Figures 5A, B**), along with diminished MMP, mtDNA levels, basal respiration, ATP synthesis, and maximal respiration levels (all *P* < 0.01; **Figures 5C–H**), suggesting that hyperthermia triggered oxidative stress and mitochondrial dysfunction in A549 cells. Moreover, hyperthermia-induced oxidative stress and mitochondrial dysfunction were strikingly suppressed by overexpression of HSP70 but were driven by knockdown of HSP70 (all *P* < 0.05; **Figures 5A–H**). Furthermore, P-gp knockdown partially abolished the repressive effects of HSP70 overexpression on hyperthermia-induced oxidative stress and mitochondrial dysfunction (all *P* < 0.05; **Figures 5A–H**). Altogether, these findings indicate that HSP70 suppresses oxidative stress and mitochondrial dysfunction by up-regulating P-gp.

### HSP70 mediates P-gp SUMO modification, thereby inhibiting ferroptosis to promote thermoresistance and *in vivo* growth of NSCLC

**Figure 6:**
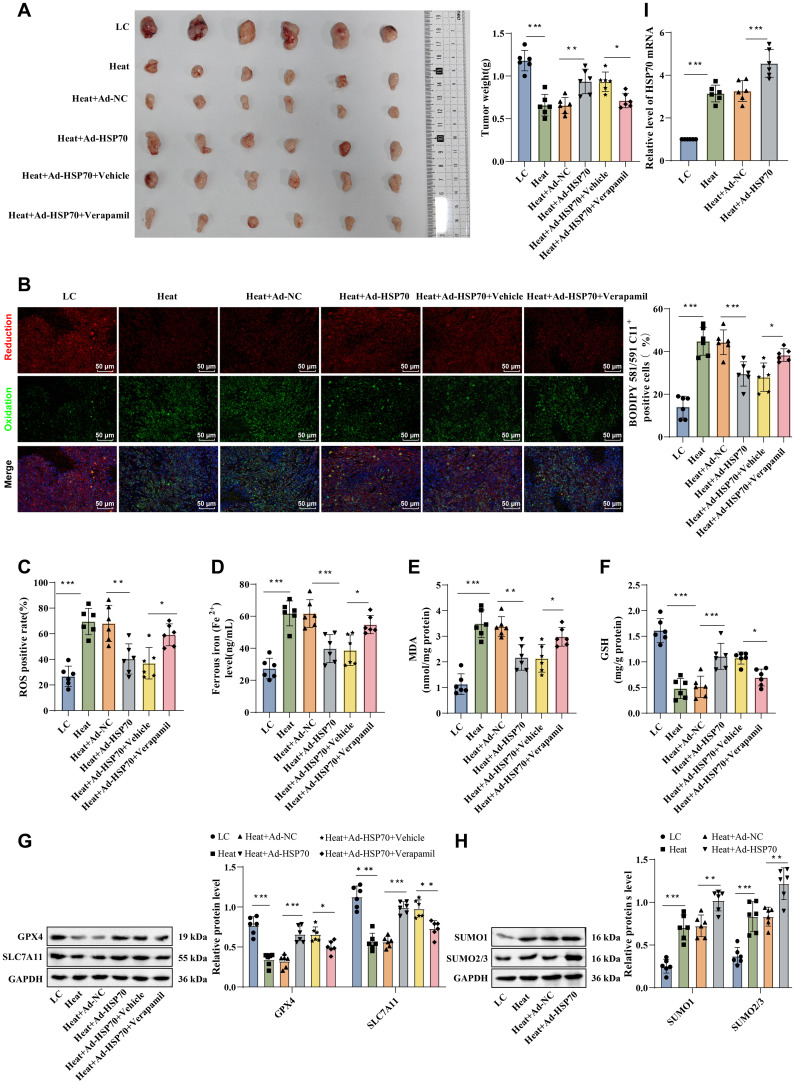
HSP70-mediated SUMOylation regulates P-gp to inhibit ferroptosis and thus boost resistance to hyperthermia and tumor growth *in vivo*. **(A)** Tumor images and weights; **(B)** Lipid peroxidation assessed by BODIPY staining; **(C–F)** Intratumoral ROS, Fe^2+^, MDA, and GSH levels; **(G, H)** Expression of ferroptosis-related proteins (GPX4 and SLC7A11), SUMO1, and SUMO2/3 detected by western blot; **(I)** HSP70 level in tumor tissues measured by RT-qPCR. n = 6. Data were presented as mean ± SD. Comparisons among groups were performed using one-way ANOVA, followed by Tukey’s multiple comparisons test. *P < 0.05, **P < 0.01, ***P < 0.001.

**Figure 7:**
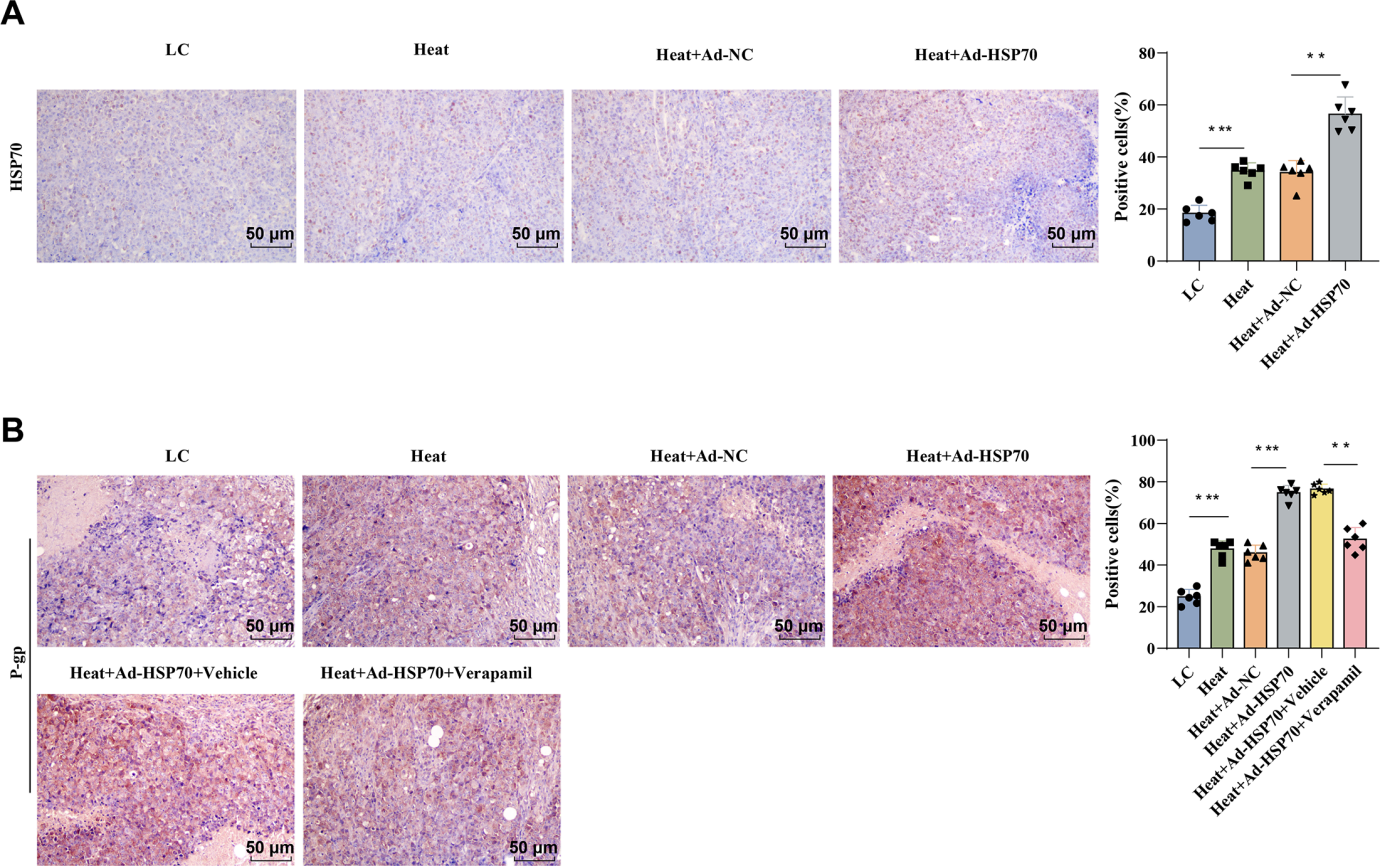
HSP70-mediated SUMOylation modulates P-gp *in vivo*. **(A, B)** IHC analysis of HSP70-positive cells and P-gp-positive cells in nude mouse tumor tissues. n = 6. Data were presented as mean ± SD. Comparisons among groups were performed using one-way ANOVA, followed by Tukey’s multiple comparisons test. *P < 0.05, **P < 0.01, ***P < 0.001.

Finally, to verify the antitumor effects and ferroptosis-inducing properties of hyperthermia *in vivo*, an *in vivo* tumor formation model of LC was established by subcutaneous inoculation of 2 × 10^6^ A549 cells into the right axillary region of BALB/c nude mice prior to hyperthermia treatment. Compared with the LC group, the Heat group had reduced tumor weight (*P* < 0.01; **Figure 6A**), highlighting that hyperthermia obviously hindered the growth of LC *in vivo*. Meanwhile, hyperthermia treatment increased lipid peroxidation, ROS, Fe^2+^, and MDA concentrations, and decreased GSH, GPX4, and SLC7A11 concentrations in mouse tumor tissues (all *P* < 0.001; **Figures 6B–G**). Hyperthermia therapy also resulted in elevated levels of HSP70, P-gp, SUMO1, and SUMO2/3 proteins in tumor tissues (all *P* < 0.05; **Figures 6H, I**, **7A, B**). These *in vivo* findings are consistent with the results of *in vitro* experiments, confirming that hyperthermia represses LC growth by inducing ferroptosis while activating HSP70-mediated SUMOylation.

Subsequently, to confirm the role of HSP70 in regulating the resistance to hyperthermia *in vivo*, we infected A549 cells with an adenoviral vector overexpressing HSP70 (Ad-HSP70) and then inoculated these cells into nude mice. The results revealed that in contrast to the Heat + Ad-NC group, the Heat + Ad-HSP70 group displayed further increases in HSP70, P-gp, SUMO1, and SUMO2/3 expression, accompanied by reduced lipid peroxidation, decreased Fe^2+^ and MDA levels, strengthened SLC7A11, GSH, and GPX4 levels, and increased tumor weight (all *P* < 0.001; **Figures 6A–I**, **7A, B**), indicating that overexpression of HSP70 depressed ferroptosis and potentiated tumor growth *in vivo*, greatly abrogating the antitumor effects of hyperthermia. In addition, to clarify the key role of P-gp in HSP70-mediated thermoresistance, the P-gp inhibitor verapamil was additionally used before hyperthermia treatment. The findings revealed that compared with the Heat + Ad-HSP70 + Vehicle group, treatment with verapamil (Heat + Ad-HSP70 + Verapamil group) reduced P-gp expression, enhanced lipid peroxidation, increased MDA, ROS, Fe^2+^ levels, decreased GSH, down-regulated GPX4 and SLC7A11, and significantly reduced tumor weight (**Figures 6A-I**, **7A, B**), illustrating that inhibition of P-gp partially counteracted thermoresistance caused by HSP70 overexpression, restoring hyperthermia-induced ferroptosis and antitumor effects. Together, these results demonstrate that HSP70 impedes ferroptosis by mediating the SUMOylation of P-gp, therefore facilitating thermoresistance and *in vivo* tumor growth in NSCLC.

## Discussion

As a well-characterized thermoprotective protein, HSP70 enhances cellular thermoresistance, a vital determinant for organismal survival responding to extreme temperatures (31). Functionally, HSP70 confers the thermoresistance of cancer cells by counteracting heat-initiated apoptosis, the predominant pathway underlying hyperthermia-induced cell demise (32). Nevertheless, there is a lack of sufficient evidence supporting HSP70-mediated thermotolerance through ferroptosis regulation in NSCLC, and the underlying mechanisms remain unreported. Our present work demonstrates that HSP70 suppresses ferroptosis by governing the SUMOylation of P-gp, ultimately promoting thermoresistance in NSCLC.

Accumulating studies reveal that heat exposure induces HSP70 expression at both the mRNA and protein levels (33–35). Cells with overexpressed HSP70 exhibit high thermotolerance by inhibiting ROS generation, reducing death receptor expression, and attenuating mitochondrial signaling cascades, thereby effectively protecting against heat-induced cell death (36). HSP70 is known as a multi-functional driver of tumorigenesis. It serves as a central regulator of cellular stress response by mediating mitochondrial homeostasis, autophagy, oxidative stress, and ferroptosis. For instance, genetic or therapeutic HSP70 ablation compromises mitochondrial dynamics and promotes autophagy in pancreatic cancer (37). HSP70 is a negative regulator of autophagy in NSCLC cells, and inhibition of HSP70 may disrupt the resistance of refractory tumor cells to conventional therapies (38). HSP70 and Bim form a complex by recruiting parkin and TOMM20, synergetically driving mitophagy (39). Furthermore, the disruption of HIF-1α/HSP70 confers the reversal of drug resistance in 5-FU-resistant hepatocellular carcinoma cells (40). Prior research has further shown that HSP70 can initiate epithelial-mesenchymal transition in LC cells and enhance their migratory and invasive capabilities (41). In addition, members of the HSP70 family have been reported to increase resistance to ferroptosis by enhancing GPX4 expression and its antioxidant function, while inhibiting lipid ROS production (42). HSPA5, also referred to as GRP78 or BiP, is a significant constituent of the HSP70 family. Its overexpression enhances GPX4 production and activity, thereby protecting glioma cells from ferroptosis by neutralizing dihydroartemisinin-induced lipid peroxidation (43). In line with these reports, our *in vitro* findings showed the tumor-promoting action of HSP70 overexpression in NSCLC cells. Importantly, HSP70 knockdown can promote ferroptosis induced by hyperthermia and enhance the anti-tumor effect of hyperthermia in NSCLC cells. Mechanistically, the ferroptosis activator Erastin can limit GPX4 expression, induce ferroptosis, and reduce radioresistance (44). Consistently, our further investigations showed that ferroptosis activation attenuated HSP70-mediated thermotolerance in NSCLC cells. This suggests that ferroptosis is a core downstream mechanism for HSP70-mediated thermotolerance of NSCLC cells.

P-gp is the most extensively researched ABC transporter linked to cancer treatment resistance (45). Hyperthermia induces HSP70 expression, which in turn up-regulates P-gp in resistant cells, thereby conferring protection against thermal stress and pharmacological agents (46). A key finding of this study is the mechanistic link between HSP70 and P-gp stability. We confirmed an interaction between HSP70 and P-gp and showed that P-gp was a target of HSP70-mediated SUMOylation. Importantly, HSP70 reduces P-gp degradation and enhances its protein stability through SUMOylation involving SUMO1/2/3, which is a crucial modification engaged in the regulation of ferroptosis (47, 48). Recent evidence has linked P-gp expression to the suppression of ferroptosis (23), a finding strongly supported by our data. Heat intervention alone triggered ROS accumulation, SOD depletion, and impaired mitochondrial function (49). The current work demonstrated that P-gp knockdown effectively nullified the repressive effect of HSP70 overexpression on ferroptosis and restored the ferroptotic response induced by hyperthermia, corresponding to changes in MMP, mtDNA levels, basal respiration, ATP synthesis, and maximal respiration levels. It is reasonable to conclude that P-gp is a key downstream molecule in HSP70-mediated thermoresistance. This aligns with prior evidence that P-gp dysfunction can provoke mitochondrial stress and ROS generation (24), while P-gp inhibition elevates mitochondrial activity, increases ATP and ROS production in the MDA-MB-231 cell line, and ultimately induces cell death (24). Together, HSP70 inhibits ferroptosis, oxidative stress, and mitochondrial dysfunction by up-regulating P-gp.

Several *in vivo* and *in vitro* studies have shown that increased HSP70 expression promotes both tumor growth and migration of different tumor cells (50–52) and that elevated HSP70 correlates with enhanced thermotolerance in a murine model of exertional heat stroke (53). Consistently, our *in vivo* experiments demonstrated that HSP70 upregulation significantly boosted tumor growth and conferred resistance to hyperthermia. This effect could be reversed by inhibition of P-gp by Verapamil, highlighting that HSP70 mediates P-gp SUMOylation to suppress resistance to hyperthermia, enhancing the anti-tumor activity of hyperthermia. Likewise, our prior study reported that HSP70 impeded ferroptosis in LC cells through SUMOylation (25). However, the prior study focused on the regulation of ferroptosis *via* SUMOylation following radiofrequency ablation, with the major findings displayed that HSP70-driven SUMOylation of HIF-1α could block the ferroptotic pathway and consequently enhance LC recurrence post-ablation. The present study demonstrated for the first time that P-gp was a direct target of HSP70-governed SUMOylation. Through this modification, HSP70 suppressed oxidative stress and mitochondrial dysfunction to restrain ferroptosis, ultimately conferring thermotolerance in NSCLC cells. These two studies revealed two distinct functions of HSP70 in response to tumor stress: it drives long-term tumor relapse and short-term thermoresistance *via* separate downstream axes. The core innovation of this study lies in its first-ever demonstration that P-gp acts as a direct target of HSP70-mediated SUMOylation and participates in regulating the molecular mechanisms underlying tumor thermotolerance. This finding not only broadens the known HSP70 regulatory cascade but also, together with the preceding finding, refines the regulatory patterns of HSP70 across different stages of tumor stress. Accordingly, our study offers a theoretical foundation for the development of targeted therapeutic strategies tailored to various clinical scenarios.

In conclusion, this study is the first to demonstrate that HSP70 enhances thermoresistance in NSCLC cells through mediating the SUMOylation of P-gp, thereby mitigating oxidative stress and mitochondrial dysfunction and ultimately suppressing ferroptosis. Mounting evidence indicates that hyperthermia profoundly modulates anti-tumor immunity and may synergistically improve the efficacy of immune checkpoint inhibitors. Mild systemic hyperthermia (39 °C-41 °C) mimics physiological fever to stimulate immune cell activation and accelerate cellular metabolism, while ablative hyperthermia exceeding 50 °C triggers the release of tumor-associated antigens and potentiates immune reactions (54). Furthermore, ferroptosis is closely associated with oxidative stress and mitochondrial dysfunction, jointly constituting a key mechanism underlying tumor immune evasion (55, 56). The present study elaborated that HSP70-mediated SUMOylation upregulates P-gp to block ferroptosis, conferring thermoresistance in NSCLC cells. Furthermore, the HSP70-P-gp axis identified herein plays a crucial role in thermoresistance in NSCLC, providing new theoretical support and potential targets for the clinical combination of thermotherapy and immunotherapy. However, certain limitations should be acknowledged. The present study was conducted in a single cell line, limiting the generalizability of our findings. Due to funding constraints and time limitations, we are currently unable to include additional lung cancer cell lines for further validation. In follow-up investigations, we will validate our findings across additional lung cancer cell lines *via* targeted manipulation of HSP70 (overexpression and knockdown). Subsequent functional assessments will include multiple ferroptosis-associated markers, such as intracellular Fe^2+^ accumulation, lipid ROS generation, as well as the expression profiling and functional characterization of GPX4, SLC7A11, and P-gp. Moreover, the precise P-gp SUMOylation sites regulated by HSP70 and other potential pathways by which HSP70 modulates ferroptosis remain to be elucidated in the following studies.

## Data Availability

The raw data supporting the conclusions of this article will be made available by the authors, without undue reservation.

## Supplementary material

Supplementary Figure 1 Cytotoxicity assay following plasmid transfection.
